# Serum levels of glucose, thyroid stimulating hormone, and free thyroxine in boys diagnosed with attention deficit hyperactivity disorder: a cross-sectional pilot study

**DOI:** 10.1186/s12883-024-03563-w

**Published:** 2024-02-26

**Authors:** Tanja Lukovac, Olivera Aleksić Hil, Milka Popović, Tatjana Savić, Aleksandra M. Pavlović, Dragan Pavlović

**Affiliations:** 1 Center for Speech and Language Pathology Higia Logos, Mirijevski Bulevar 17 b, Belgrade, 11000 Serbia; 2 Institute of Mental Health, Palmoticeva 37, Belgrade, 11000 Serbia; 3 Beo-Lab Laboratories, Resavska 58-60, Belgrade, Serbia; 4 Institute for Biological Research “Siniša Stanković” - National Institute of the Republic of Serbia, University of Belgrade, 142 Despot Stefan Boulevard, Belgrade, Serbia; 5 Faculty of Special Education and Rehabilitation, University of Belgrade, Visokog Stevana 2, Belgrade, 11000 Serbia

**Keywords:** FT4, TSH, Glucose, ADHD, Neurodevelopmental disorders, Children, Boys

## Abstract

**Background:**

Although attention deficit hyperactivity disorder (ADHD) is a common neurodevelopmental disorder, its aetiology remains unclear. We aimed to establish a relationship between ADHD diagnosis and serum levels of glucose, free thyroxine (FT4), and thyroid stimulating hormone (TSH) in primary school aged boys.

**Methods:**

In a cross-sectional study, we enrolled 133 participants aged 6.5–12.5 years, 67 of whom met DSM-5 criteria for ADHD and 66 healthy age-matched boys. The ADHDT test (ADHDT) was used to assess ADHD symptoms and the Wechsler Intelligence Scale for Children – Revised was used to exclude participants with cognitive deficits. The ADHD participants were tested using the Iowa Conners’ Teacher Rating Scale.

**Results:**

The ADHD participants had lower glucose levels, higher TSH values, and significantly lower FT4 values than the control group. The multiple logistic regression analysis showed that TSH is a parameter that is 2.7% more likely to occur in the ADHD group. We found a significant correlation between the TSH level and the symptoms of hyperactivity (*r* = 0.318, *p* = 0.009) and impulsivity (*r* = 0.275, *p* = 0.024) as well as between the glucose level and the symptoms of hyperactivity (*r* = 0.312, *p* = 0.010).

**Conclusions:**

Certain ADHD symptoms may correlate with certain hormonal patterns. Our results suggest that the likelihood of suffering from ADHD was lower when FT4 levels were elevated. One biochemical parameter that was significantly and independently associated with the diagnosis of ADHD was the serum TSH level.

**Trial registration:**

On June 26, 2018, at its VI session in 2018, the Ethics Committee of the Institute for Mental Health in Belgrade, Serbia, has considered and unanimously approved the conduct of the research, under the number 1704/1.

## Background

Attention deficit hyperactivity disorder (ADHD) is one of the most common neurodevelopmental disorders, affecting 5% of children and adolescents and 2.8% of adults worldwide [1, 2]. ADHD is more common in males than females, with a 3:1 ratio in population studies [3, 4]. The phenotypic variability and severity of ADHD is very heterogeneous and is categorised into three groups according to the *Diagnostic and Statistical Manual of Mental Disorders, Fifth Edition*: inattentive, impulsive/hyperactive and combined [5]. The identification of ADHD symptoms is usually based on the use of specific tests, such as the Conners’ Teacher Rating Scale [6] and the Attention Deficit Hyperactivity Disorder Test (ADHDT) [7].

The aetiology of the disorder is probably multifactorial and person dependent, which makes it difficult to identify and describe the underlying biological mechanisms. ADHD is thought to be caused by an interplay of genetic susceptibility [8, 9] and exposure to numerous pre- and postnatal environmental risk factors, including toxins [10], maternal smoking and alcohol consumption [11], low birth weight [12] and various socio-psychological factors [13]. In addition, recent research has suggested the role of endocrinological factors in the pathogenesis of ADHD. Although there is ample evidence of a link between improved mental performance and subjects’ blood glucose levels, the effects of blood glucose levels on cognition and psychological processes remain enigmatic [14]. A study by Lindblad et al. [15] found a corellation between ADHD and blood glucose levels. The authors found that the average 3-month blood glucose levels were significantly higher in the ADHD group compared to the control group, while fasting blood glucose was similar in the ADHD group and the control group [15]. The link between ADHD and diabetes mellitus is also recognised and is attributed to as yet unknown genetic factors [16, 17]. The prevalence of ADHD is 40% higher in children with type 1 diabetes [18]. In addition, a dual diagnosis of diabetes and ADHD in childhood is associated with poorer diabetes control [18].

Thyroid hormones play a vital role in neural development by influencing the differentiation and migration of neurons, synapse formation and myelination [19]. Several studies have shown that endocrine disorders, especially thyroid abnormalities, can be associated with ADHD [20, 21]. Thyroid deficiency during embryonic development leads to irreversible impairment of cognitive and motor functions [22, 23]. In addition, the manifestations of hyperthyroidism (anxiety, hyperirritability, inattention and hyperactivity) are very similar to the symptoms of ADHD. However, some studies investigating this relationship have come to contradictory conclusions [21, 24]. One of the first studies [21, 22] to explore the relationship between ADHD and thyroid hormone levels concluded that the prevalence of thyroid dysregulation in ADHD individuals was 5.2%, compared to < 1% in the general population [25]. Zader et al. [25] showed that children with hyperthyroidism are twice as likely to be diagnosed with ADHD, while Álvarez-Pedrerol et al. [26] found no association between thyroid hormone levels and the underlying neurophysical symptoms of ADHD. There is also solid evidence that high levels of thyroid stimulating hormone (TSH) and low levels of free thyroxine (FT4) are associated with ADHD symptoms, even when levels are within the normal range [26]. Thyroid hormones are also responsible for regulating glucose homeostasis and act as insulin agonists and antagonists [27].

The aim of this study was to investigate the baseline endocrinological status in a cohort of primary school boys diagnosed with ADHD using readily available screening tests such as serum levels of glucose, TSH and FT4 and their association with the clinical presentation of ADHD. We hypothesised that children with ADHD will have higher serum glucose and TSH levels and lower FT4 levels compared to age-matched children without an ADHD diagnosis.

## Methods

### Study design

**Fig. 1:**
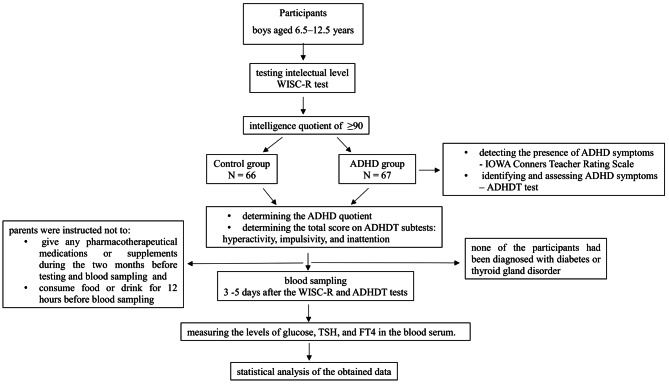
Study flow chart illustrates the sequential steps undertaken in the cross-sectional study conducted at the Institute of Mental Health in Belgrade, Serbia, examining the relationship between ADHD, biochemical parameters, and cognitive assessments in boys aged 6.5–12.5 years

We conducted a cross-sectional study at the Institute of Mental Health in Belgrade, Serbia (Fig. 1). Two groups of boys aged 6.5–12.5 years participated in the study: an ADHD group and a control group. The groups were matched based on predictive and sociodemographic variables. Male subjects were selected to increase the homogeneity of the data set and to take into account the prevalence of ADHD in males. The participants came from the same socioeconomic background and from families with neurotypical development of other siblings. Global intellectual level was tested using the Wechsler Intelligence Scale for Children – Revised (WISC-R) test [28]. Participants with a below-average intellectual level were excluded from the study. The diagnosis of ADHD was made by a psychiatrist, an expert in the field of child psychiatry. All tests were conducted at the Institute of Mental Health in Belgrade, Serbia. The IOWA Conners Teacher Rating Scale was used to determine the presence of ADHD symptoms [6]. The ADHDT was used to determine and rate the severity of ADHD symptoms [7]. The total scores of the ADHDT subtests for hyperactivity, impulsivity and inattention symptoms were obtained for all participants in both groups and the mean scores were included and analysed. The ADHD quotient was determined in both groups. The WISC-R and ADHDT tests were performed 3–5 days prior to blood sampling. The ADHDT (Gilliam 1995) was used to assess the severity of ADHD symptoms. Parents of subjects were instructed not to administer pharmacotherapeutic medications or supplements in the two months before testing and blood sampling, while none of the participants had been diagnosed with diabetes or thyroid disease. The participants were instructed not to consume any food or drink 12 h before the blood sample was taken. Blood sampling was performed at the Institute of Mental Health and serum samples were stored according to standard operating procedures and transported to the Beo-lab laboratory for further analysis.The study lasted eight months, from October 2019 to May 2020.

### Participants

The sample size was calculated using G*power 3.1.9.4; with an effect size of 0.3, an α error probability of 0.05 and a study power of 0.95. The total sample size for determining the difference between the groups for independent samples was 99. This study included 133 boys, 66 in the control group and 67 in the ADHD group. Participants in both groups had an intelligence quotient of ≥ 90, according to the WISC-R. The inclusion criteria for the ADHD group are: Age 6 to 12.5 years, presence of ADD/ADHD, normal cognitive development, consistent physical activity, balanced diet, willingness to cooperate. The diagnosis of the disorder is made by a paediatric neuropsychiatrist on the basis of the diagnostic criteria of the DSM-5 classification. The diagnosis is clinical and based on a comprehensive medical, developmental, educational and psychological assessment, which additionally uses standardised assessment tools such as the Iowa Conners Rating Scale questionnaire. The exclusion criteria for the study were: Presence of neurological disorders, other psychiatric disorders, somatic disorders, developmental coordination disorders, visual or hearing impairments, diabetes, motor disabilities, visuospatial dysgnosia, stress exposure.

### ADHD group

The ADHD group consisted of 67 participants diagnosed with ADHD at the Institute of Mental Health in Belgrade, Serbia. Boys with comorbidities were excluded from the study.

### Control group

Sixty-six participants from the general population voluntarily participated in the study with the written consent of their parents. Family history showed that no developmental problems, including ADHD, were present.

### Procedures

Experimental testing began with a brief interview with parents and participants and an assessment of the child’s daily routines, diet and supplementation. All participants underwent the WISC-R and the ADHDT to determine intellectual status and assess the type of ADHD. The ADHDT (Gilliam 1995) is a 36-item questionnaire based on the Diagnostic and Statistical Manual of Mental Disorders (DSM-4) criteria for ADHD. It is divided into three subtest (hyperactivity, inattention and impulsivity). Each item is assigned a standardised score (0 – no problem, 1 – mild problem, 2 – severe problem). The final test score is compared with a normative table to determine the severity of ADHD symptoms. The hyperactivity questionnaire (13 items) assesses the child’s speech and body movements. The impulsivity questionnaire (10 questions) describes how the child behaves when waiting for his/her turn, when playing a game with fixed rules, when talking or when making decisions. Finally, the inattention questionnaire (13 questions) examines the child’s concentration on certain tasks, the completion of tasks, planning and organising skills. The assessment was conducted in a single session, with breaks taken to prevent fatigue.

Blood was drawn three to five days after the interview. On the day of blood sampling, participants were prepared for the procedure by being given relevant information to minimise their anxiety. Fasting venous blood was drawn at 9.00 am. The laboratory materials required for the collection and storage of the blood were provided and the blood tests were performed in the same laboratory using identical methods for all participants.

### Measurements

The blood samples were taken in a biochemical test tube with a minimum gel volume of 4 mL. The blood samples were allowed to rest for 15 min due to coagulation and then centrifuged for 10 min at 1800 g (approximately 3000 rpm). After separation, the serum samples were stored at -20 °C and then analysed. The levels of glucose, TSH and FT4 were measured in the blood serum.

The quantitative determination of glucose in human serum, free of haemolysis and bacterial contamination and without the addition of preservatives, was carried out using the Beckman Coulter AU400 Chemistry Analyser, Brea California, USA. The glycemic reference values for children are up to 6.1 mmol/l after fasting.

We used the Immunoassay System Access, Beckan Coulter, Brea, California, USA, for the quantification of human TSH and FT4 levels in human serum and plasma. The expected blood TSH and FT4 levels in children’s blood are 0.54–4.53 mIU/L and 12.0–22.0 pmol/L, respectively.

### Statistical analysis

The Kolmogorov–Smirnov test was used to assess the normal distribution of the data. A one-way analysis of variance (ANOVA) was used to compare the measured biochemical parameters and symptoms (hyperactivity, impulsivity and inattention) in the control and ADHD groups. Pearson’s correlation coefficient was used to evaluate the association between the test scores (WISC-R and ADHDT), hyperactivity, impulsivity and inattention with the biochemical parameters of the ADHD participants. A multiple logistic regression model was used to understand the functional relationship between the biochemical parameters and ADHD diagnosis. All statistical analyses were performed using SPSS statistical software (version 20.0.0, IBM Corp., Armonk, New York, USA) and STATISTICA statistical software (version 8.0, StatSoft, Inc., Tulsa, USA).

## Results

The 67 participants with ADHD were between 6.5 and12.5 years old and had an average age of 10.13 ± 1.41 years. The 66 participants in the control group aged 6.5–12.5 years had an average age of 9.94 ± 1.52 years. The participants in the two groups did not differ significantly in age (t = − 0.756, df = 131, *p* > 0.05).

The ADHD T-score of the experimental group ranged from a maximum value of 126 to a minimum value of 60, while the control group had a range from 121 to a minimum value of 13. In contrast to the control group, in which the values for hyperactivity, inattentiveness and impulsivity were a maximum of 16 and a minimum of 0 the values for hyperactivity ranged from a maximum of 8 to a minimum of 2, for impulsivity from a maximum of 16 to a minimum of 2 and for inattention from a maximum of 19 to a minimum of 2.

A significant difference between the results of the ADHD group and the control group was found in the results of the ADHDT and the WISC-R. The results of the symptoms measured in the subtests are given as mean ± standard deviation (SD) and are listed in Table 1.

**Table 1:** Mean values of the symptoms measured for the control and ADHD groups, ADHDT test results

	Control group, *n* = 66		ADHD group, *n* = 67
Hyperactivity, mean ± SD (range)	5.05 ± 3.60 (0–16)	9.82 ± 3.33 (4–18)	
Impulsivity, mean ± SD (range)	5.00 ± 3.67 (0–14)	10.31 ± 3.43 (2–16)	
Inattention, mean ± SD (range)	4.00 ± 2.88 (0–11)	9.00 ± 3.39 (2–19)	

ADHD, attention deficit hyperactivity disorder; ADHDT - Attention Deficit Hyperactivity Disorder Test

The scores for hyperactivity, impulsivity and inattention were twice as high in the participants with ADHD as in the control group. In addition, the one-way ANOVA confirmed that hyperactivity, impulsivity and inattention were significantly higher in the ADHD group than in the control group (F_0.001, 1, 131_ = 63.236; *p* = 7.503E-13. F_0.001, 1, 131_ = 74.506; *p* = 1.776E-14. F_0.001, 1, 131_ = 83.889; *p* = 8.882E-16 respectively).

The results of the analysed biochemical parameters analyzed are expressed as mean ± SD and are shown in Table 2.

**Table 2:** Mean values of serum glucose, TSH and FT4 levels for the control and ADHD groups

	Control group, *n* = 66	ADHD group, *n* = 67
Glu (mmol/L), mean ± SD (range)	5.04 ± 0.45 (3.6–6.7)	4.99 ± 0.51 (3.2–6.4)
TSH (µIU/mL), mean ± SD (range)	2.60 ± 1.08 (1.12–6.98)	2.73 ± 1.18 (0.88–6.88)
FT4 (pmol/L), mean ± SD (range)	13.46 ± 2.52 (9.2–19.6)	12.63 ± 1.98 (9.38-19)

ADHD, attention deficit hyperactivity disorder; FT4, free thyroxine; TSH, thyroid stimulating hormone

The mean values for serum glucose, TSH and FT4 were not above the normal range in either group. One-way ANOVA revealed significantly lower FT4 levels in the participants with ADHD than in the control subjects (F_0.05, 1, 131_ = 4.449; *p* = 0.037). Serum levels of glucose and TSH did not differ significantly between the control and ADHD groups (F_0.05, 1, 131_ = 0.39; *p* = 0.533 and F_0.05, 1, 131_ = 0.422; *p* = 0.517, respectively). Participants diagnosed with ADHD had slightly lower glucose levels and slightly higher TSH levels than the control group. The serum levels of TSH and FT4 were significantly negatively correlated in the control group (*r* = -0.38, *p* = 0.002), while in the ADHD group the levels of TSH and FT4 were negatively correlated, but without significance (*r* = -0.19, *p* = 0.13).

Analysis using Pearson correlation analysis (Table 3) revealed a non-significant negative correlation between serum FT4 levels and ADHDT and WISC-R scores for all participants diagnosed with ADHD. Serum glucose levels showed a significant positive correlation with hyperactivity. A significant positive correlation was found between serum TSH levels, hyperactivity and impulsivity.

**Table 3:** Pearson correlation coefficient between the scores of ADHDT, hyperactivity, impulsivity, inattention, WISC-R and biochemical parameters

	ADHD T		Hyperactivity		Impulsivity		Inattention		REVISK					
									IQ verbal		IQ non verbal		IQ total	
	r	*p*	r	*p*	r	*p*	r	*p*	r	*p*	r	*p*	r	*p*
Glu (mmol/l)	0.025	0.841	0.312	0.01^**^	0.225	0.068	-0.089	0.472	0.13	0.293	0.066	0.593	0.128	0.302
TSH (µIU/ml)	0.075	0.548	0.318	0.009^**^	0.275	0.024^*^	-0.14	0.258	0.004	0.973	-0.13	0.293	-0.068	0.588
FT4 (pmol/l)	-0.014	0.909	-0.141	0.254	-0.151	0.224	-0.142	0.252	-0.096	0.441	-0.125	0.313	-0.1	0.422

ADHD, attention deficit hyperactivity disorder; ADHT-T, attention deficit hyperactivity disorder test, FT4, free thyroxine; IQ, intelligence quotient; TSH, thyroid stimulating hormone; WISC-R, Wechsler Intelligence Scale for Children – Revised

The multiple logistic regression model analysis showed that TSH is a biochemical parameter associated with the diagnosis of ADHD (Table 4). Compared to the control group, TSH was 1.027 higher in boys with ADHD, i.e. the probability of the outcome occuring is 2.7% higher in the ADHD group.

**Table 4:** Results of the multiple logistic regression model for the ADHD group compared to the control group

	B	SE	Wald χ^2^ test	df	p	Exp(B)
Glucose (mmol/L)	-0.369	0.393	0.880	1	0.348	0.692
TSH (mIU/L)	0.027	0.165	0.027	1	0.870	1.027
FT4 (pmol/L)	-0.173	0.085	4.113	1	0.043	0.841

ADHD, attention deficit hyperactivity disorder; FT4, free thyroxine; SE, standard error; TSH, thyroid stimulating hormone; df, degrees of freedom; Exp(B), exponentiation of the B coefficient

FT4 had significantly lower odds ratios in boys with ADHD (0.841) than in boys without ADHD. The negative coefficient value for FT4 indicate that the probability of being diagnosed with ADHD is lower when FT4 levels was elevated.

## Discussion

The results of our study show that FT4 levels are significantly lower in children diagnosed with ADHD compared to the control. In addition, a significant positive correlation was found between serum glucose levels and hyperactivity, while serum TSH levels were significantly correlated with hyperactivity and impulsivity.

To the best of our knowledge, this is the first experimental study on hormonal status and ADHD conducted in Eastern Europe. Although an association between ADHD and thyroid gland disorders has been established, there is no strong evidence for a casual relationship [24]. Therefore, in our study, we investigated the association between glucose, FT4 and TSH levels and ADHD diagnosis in children and the association of the hormonal status with the sympthoms of hyperactivity, impulsivity and inattention.

There are no specific biochemical, electrophysiological or imaging biomarkers for ADHD, which makes diagnosis and research into this disorder challenging. Published research on ADHD has mainly focused on the clinical profile, the influence of sociodemographic status and the comorbidities of the disorder [29, 30]. The hormonal influence on the diagnosis and presentation of ADHD has been suggested but is still not fully understood [31, 32]. Most studies have attempted to find an association between ADHD and sugar consumption, but the results have been conflicting. Studies by Azadbakhy and Esmaillzadeh [33] and Lien et al. [34] showed a possible positive association between ADHD and sugar-rich food consumption, whereas Kim and Chang [35] did not find this association. Lindblad et al. [15] reported an association between ADHD and impaired blood glucose homeostasis. In our study, serum glucose levels were slightly lower in participants diagnosed with ADHD and correlated positively only with symptoms of hyperactivity. In this study, a negative coefficient value was found in the multiple logistic regression model for glucose. Given the positive correlation we found between hyperactivity and glucose levels, we conclude that further research should be conducted to investigate the relationship between glucose levels and specific symptoms of ADHD.

The relationship between ADHD and insulin resistance (IR) is the subject of ongoing research and the link between these two conditions is not yet fully understood. Our study found a positive correlation between serum glucose levels and hyperactivity. Other studies suggest a possible link between ADHD and other factors related to IR [36, 37]. This may be due to common risk factors (e.g. obesity, lack of exercise and abnormal dietary habits) or the possible metabolic effects of ADHD stimulants (such as methylphenidate). While there is some evidence suggesting a link between ADHD and IR, direct and consistent evidence is limited. The study by di Girolamo et al. [37] examines the relationship between ADHD and metabolic syndrome (MS) in adults. The study, conducted in Italy, examines the role of MS components and IR indices in this population. The results show that 10.8% of the ADHD patients analysed met the criteria for MS. The study emphasises the importance of considering individual MS components, with blood triglyceride levels identified as the most important predictor of MS in adult ADHD patients. This suggests that lifestyle factors, including unhealthy dietary habits and obesity, may contribute to the prevalence of MS and IR in individuals with ADHD.

Thyroid hormones regulate systemic glucose metabolism and may also be involved in the regulation of glucose metabolism in the brain [38]. Thyroid hormones are important for cognitive function in children and adults [39], and they also modulate memory and attention [40, 41]. Previous studies have indicated that TSH concentrations are significantly lower in children with ADHD than in children without ADHD [24, 42–44]. However, our study showed that TSH levels were slightly higher in male participants diagnosed with ADHD than in controls, with a 2.7% increase in the odds of an ADHD diagnosis in participants with higher serum TSH levels.

Some studies indicate a link between low TSH levels in newborns and the later onset of ADHD and neurodevelopmental disorders [45–47]. However, several studies have shown that children with ADHD symptoms do not have a significantly different thyroid hormone profile than children without ADHD symptoms [48, 49].

Our results suggest that certain ADHD symptoms may be associated with the respective hormonal patterns. We found that the prevalence of hyperactive and impulsive symptoms of ADHD is higher in children with higher serum TSH levels. The observed association between serum TSH levels and ADHD symptoms in children raises interesting questions about the possible mechanisms underlying this association. Several factors could contribute to the link between high TSH levels and ADHD symptoms. Thyroid hormones, including TSH, play a critical role in neurodevelopment, particularly in the development of the foetal brain. Previous studies have shown that maternal thyroid dysfunction may increase the risk of ADHD in the offspring [50]. The current findings may suggest that disturbances in thyroid function during early development or infancy may affect the neural pathways associated with ADHD symptoms. In addition, TSH influences the regulation of neurotransmitters, including dopamine and norepinephrine, which play a role in ADHD [51]. Dysregulation of these neurotransmitters is a key feature of ADHD, and changes in thyroid function could potentially disrupt the delicate balance and contribute to ADHD symptoms such as hyperactivity. It is also important to note that TSH affects metabolism, and disturbances in thyroid function could contribute to metabolic changes associated with ADHD behaviours [52]. Finally, thyroid dysfunction, including autoimmune thyroiditis, is associated with inflammation. Inflammation and immune system dysregulation have been suggested as factors in the development of ADHD [53], and the inflammation mediated by thyroid dysfunction could potentially contribute to ADHD symptoms. Further research is needed to decipher the complex interplay between TSH levels and ADHD symptoms and to mechanistically validate the findings presented in this study.

Kuppili et al. [44] and Albrecht et al. [24] showed that serum T4 and FT4 levels were significantly lower in children with ADHD than in children without ADHD. In our study, we found significantly lower FT4 levels in children diagnosed with ADHD than in controls. This result is consistent with the research of Álvarez-Pedrerol [26] and co-workers, who found an association between high levels of FT4 and lower risk of attention deficit symptoms. A non-significant negative correlation between lower serum FT4 levels, higher ADHD symptoms and intellectual level in subjects with ADHD in this study showed that the likelihood of having ADHD was lower when FT4 levels were increased. Interestingly, we also found that serum levels of TSH and FT4 were significantly negatively correlated in the control group but not in the ADHD group. This additional correlation analysis was performed to find out whether there are differences in the relationship between TSH and FT4 in the control and ADHD groups. The amount of TSH depends on the amount of FT4, i.e. when the amount of FT4 is low, TSH increases and the thyroid gland produces more FT4. The results obtained could indicate that the relationship between TSH and FT4 tends to be different in the ADHD group. Significantly lower FT4 levels in participants with ADHD than in controls could be the result of poor thyroid function. Thyroid hormones, including FT4 and T4, play a crucial role in the regulation of metabolism and the development of the nervous system. Hypothyroidism, a condition where the thyroid gland does not produce enough thyroid hormones, can lead to a variety of symptoms including fatigue, weight gain and cognitive impairment. However, the link between hypothyroidism and ADHD is not well established. The present findings suggest a complex relationship between biomarkers of thyroid function and ADHD. Thyroid hormones play a crucial role in the development of the nervous system, including the brain. Disruptions in thyroid function during critical periods of brain development can affect cognitive function and behaviour [52]. In addition, shared genetic factors and the intricate relationship between thyroid hormones and other hormones involved in brain development may contribute to the observed associations [24]. Further research, including longitudinal studies and additional neuronal and metabolic biomarkers, is needed to unravel the complex interactions between thyroid function and ADHD and to understand the underlying mechanisms.

### Limitations

The current study has several limitations. Only two hormonal parameters were included and measured, together with blood glucose levels. Therefore, we cannot completely exclude a possible influence of dietary intake on the results. In addition, we could not fully control whether the boys had eaten or drunk anything 12 h before the blood sample was taken. We recommend including a larger number of hormonal parameters and 3-month glucose levels in future studies to account for these limitations. A larger number of participants and the inclusion of female subjects would increase the generalisability of our results.

## Conclusions

The results of this study indicate a significant positive correlation between serum TSH levels and hyperactivity and impulsivity and between serum glucose levels and hyperactivity in boys with ADHD compared to neurotypical age-matched controls. The results also showed that the likelihood of developing ADHD was lower when FT4 levels were elevated. One biochemical parameter that was significantly and independently associated with the diagnosis of ADHD was serum TSH levels. In conclusion, our research offers important insights into the intricate connection between hormone levels, specifically thyroid function, and ADHD in children. These findings contribute to the expanding body of evidence highlighting the impact of hormones on ADHD, but it is crucial to acknowledge the multifaceted nature of this condition. Despite the challenges of diagnosing ADHD due to the absence of specific biomarkers, our study underscores the importance of conducting further research to identify reliable indicators. Moreover, the conflicting results in previous studies regarding the relationship between sugar intake and ADHD serve as a reminder of the complexity of this disorder. Future research should expand its focus to include other potential biomarkers and explore the potential interplay between genetics and environmental factors.

## Data Availability

Available at the Institute of Mental Health in Belgrade, Serbia (approval number 1704/1).
